# A Hypothetical Modelling and Experimental Design for Measuring Foraging Strategies of Animals

**DOI:** 10.3390/jintelligence10040078

**Published:** 2022-10-02

**Authors:** Ray-Ming Chen

**Affiliations:** School of Mathematics and Statistics, Baise University, Baise 533000, China; raymingchen@bsuc.cn

**Keywords:** density-driven forage, structural metric, foraging radial level, experimental design, LMRFT-based and SMRFT-based cognition

## Abstract

Based on animal long-term and short-term memory radial foraging techniques (or LMRFT and SMRFT), we devise a modelling approach that could capture the foraging behaviours of animals. In this modelling, LMRFT-based optimal foraging paths and SMRFT-based ones are constructed with respect to different levels of foraging strategies. Then, by a devised structural metric, we calculate the structural distance between these modelled optimal paths and the hypothetical real foraging paths taken by agents. We sample 20 foods positions via a chosen bivariate normal distribution for three agents. Then, we calculate their Euclidean distance matrix and their ranked matrix. Using LMRFT-based or SMRFT-based optimal foraging strategies, the optimal foraging paths are created. Then, foraging strategies are identified using optimal parameter learning techniques. Our results, based on the simulated foraging data, show that LMRFT-based foraging strategies for agent 1,2 and 3 are 3, 2 and 5, i.e., agent 3 is the most intelligent one among the three in terms of radial level. However, from the SMRFT-based perspective of strategies, their optimal foraging strategies are 5,5 and 2, respectively, i.e., agent 1 is as intelligent as agent 2 and both of them have better SMRFT-based foraging strategies than agent 3.

## 1. Introduction

### 1.1. Significance Statement

This article provides a method for measuring the optimality of animal foraging, a popular topic in behavioural ecology. The approach is a new one, while its application is wide and practical. The idea lies on a devised metric, which is used to calculate the structural distance so as to identify animal foraging strategies. The modelling approach could benefit ecological protection, social effect evaluation and environmental planning. This article will shed light on the measurement of animal behaviours via a given set of models. It will provide some practical applications for further field study.

### 1.2. Introduction

Cognitive maps are mental representations that animals form from experience and hunting foods Tolman (1948). Though their existence is highly controversial among researchers (Bennett 1996; Mackintosh 2002), some formation of foraging strategies is present in some animals Matzel et al. (2011). Despite the controversy of explicit forms of cognitive “maps”, the concept of animal cognition is widely accepted and recognised (Poirie et al. 2020; Jozefowiez et al. 2009).

Among all animal behaviours, modelling the foraging activity probably is the most important one. It involves some form of intelligence and cognitive abilities important for survival Brown and Gordon (2000). The main purpose of this study is to find a way to measure foraging strategies of animals and to design a set of implementation procedures to conduct such measurements. The idea is to place the identical foods at various positions, or alternatively (for intangible positions), to prearrange distinct foods with some properties (nutrition, edibility, duration, etc.). Then, we record these positions or properties of the corresponding foods via a Cartesian coordinate system or high-dimensional vectors. Then, we calculate the Euclidean distance matrix for the placed positions of foods. Once the foods are set, an animal is introduced and its optimal foraging path is created using the concept of LMRFT-based optimal foraging techniques and SMRFT-based ones, where LMRFT and SMRFT stand for “Long-term Memory Radial Foraging Techniques” and “Short-term Memory Radial Foraging Techniques”, respectively. Long-term or short-term memory is very important for animals in retaining their learned foraging skills or in remembering their previous foraging spatial locations (Bracis et al. 2015; Roth et al. 2012; Shaw and Annette 2020). The foraging techniques highly rely on the concept of density-based spatial clustering of applications with noise (DBSCAN), which is notably applied in data mining, knowledge discovery and clustering (Sander et al. 1998; Schubert et al. 2017). However, our main purpose is not to cluster the data, but to delve into the foraging behaviours of animals—in particular, we have to look into their relations with the environment. In other words, we have to consider the landscape ecology Wiens (2005). Nonetheless, DBSCAN does provide some fundamental ideas and mechanisms about foraging behaviours via food density, which is crucial in food searching and hunting. DBSCAN could also be applied to track the movement patterns of animals.

The density of foods affects the dynamical behaviours of animals de Knegt et al. (2007); Mellgren et al. (1984). Therefore, we cannot adopt DBSCAN at its face value—some amendments must be made. Among them, the radii for cores are regarded as a parameter for measuring the foraging strategies; the minPts (minimal number of data points) is also treated as a parameter to help identify animal foraging strategies, and the concept of optimal paths is introduced (Parker and Smith 1990). Another characteristic of our amendment is the behaviour is not static, i.e., fixed by a predetermined rule, but the dynamical domains must be taken into consideration—this reflects part of the intelligence of numeracy and memory of the simulated animals. For example, suppose the food domain, i.e., the potential foods for animal, in the previous foraging step is $FOOD_{i-1}$. The animal is now foraging at step *i*, and food *f* is eaten at this step. Then, in the next step, the food domain for the animal should be $FOOD_{i}=FOOD_{i-1}-\left\{f\right\}$. By repeating this foraging behaviour, based on LMRFT-based and SMRFT-based foraging techniques in Section 2—without predetermined knowledge of animals strategies, these techniques serve the knowledge discovery to reflect their degrees of intelligence (or radial levels) from various perspectives—the modelled optimal foraging paths are created. By identifying, via structural metric Chen (2021, 2022), the closest paths between the modelled ones and the hypothetically real ones, we obtain the optimal match of the foraging strategies for the animals. For demonstrative purpose, we choose three animals and three batches of foods—each batch contains 20 foods spread in 20 positions chosen randomly from a bivariate normal distribution. By calculating the three Euclidean distance matrices for the three batches of foods, we rank the distance matrices. Then, LMRFT-based and SMRFT-based foraging strategies are applied to yield animal optimal paths. By calculating the distances between the optimal foraging paths and the hypothetically real ones (i.e., simulated data whose role is deemed as real data), we match the foraging strategies for the three animals, respectively. The result shows, from LMRFT-based foraging perspective, animal 3 has the highest radial level. However, from SMRFT-based foraging perspective, animal 1 and 2 are better than animal 3. Our approach has several characteristics:

1. The modelling is quite comprehensive, since the utilised approach combines various theories to reflect animals’ foraging strategies: DBSCAN, foraging density theories, structural metrics, optimal theories, etc.;
2. The modelling is flexible in extracting the parameters related to specific animals foraging behaviours. Since most of the assumptions are treated as parameters, the model could be amended to fit most of the specific cases;
3. For further refinement of the modelling, the original model could be linked to or incorporated into other technique-based models: data mining, knowledge discovery, machine learning, etc.
4. This modelling provides a complete set of implementation procedures, which provide a platform for a model-based and intuitive experimental design. In particular, the adoption of various metrics would provide some visualised properties for the measurements.

## 2. Foraging Models

There are two main perspectives in quantifying the foraging strategies of animals: LMRFT and SMRFT (Bracis et al. 2015; Börger et al. 2008). We associate an animal foraging radial level, or foraging radius, with its perceptive capability for finding food. For LMRFT, the animal perceives foods from past experience and long-term learning memory Vorhees and Williams (2014). For SMRFT, animal foraging behaviour is bound to its current positions or short-term memory or learning techniques.

For any metric space (S,d), where *S* is a set and *d* s a metric on *S*, and any x∈S, define its neighbouring set (including borders) by $B^{S}\left(x;\delta \right):=\left\{y\in S:d\left(x,y\right)\leq \delta \right\}$.

**Definition** **1.** *(relative orders $\leq_{x},{<}_{x},{=}_{x},\neq_{x}$) For any x∈S, define an inequality relation $\leq_{x}$ on S by $y\leq_{x}z$ if and only if d(x,y)≤d(x,z) for all y,z∈S. Similarly, define $y{=}_{x}z$ (or $y\neq_{x}z$) if and only d(x,y)=d(x,z) (or d(x,y)≠d(x,z)). Define $y{<}_{x}z$ if and only if $y\leq_{x}z$ and $y\neq_{x}z$.*

We could observe that if (S,d) is a metric space, then $\leq_{x}$ is a total order on *S*.

**Definition** **2.** *(relative minimum $min_{x}^{S}\left(k\right)$) Define $min_{x}^{S}\left(k\right)$ as the k-th least value in the set {d(x,y):y∈S}.*

**Definition** **3.** *Define $B_{x}^{S}\left(k\right)=B^{S}\left(x;min_{x}^{S}\left(k\right)\right)$.*

**Example** **1.** *Suppose S={x≡(1,2),y≡(−2,4),p≡(0,5),q≡(3,1)} and d is the Euclidean metric. Then, $min_{x}^{S}\left(1\right)=min_{y}^{S}\left(1\right)=min_{p}^{S}\left(1\right)=min_{q}^{S}\left(1\right)=0$, $min_{x}^{S}\left(2\right)=\sqrt{5},min_{x}^{S}\left(3\right)=\sqrt{10},min_{x}^{S}\left(4\right)=\sqrt{13}$; $B_{x}^{S}\left(1\right)=\left\{x\right\},B_{x}^{S}\left(2\right)=\left\{x,q\right\},B_{x}^{S}\left(3\right)=\left\{x,p,q\right\},B_{x}^{S}\left(4\right)=\left\{x,y,p,q\right\}$; by the same manner, $min_{y}^{S}\left(2\right)=\sqrt{5},min_{y}^{S}\left(3\right)=\sqrt{13},min_{y}^{S}\left(4\right)=\sqrt{34}$; $B_{y}^{S}\left(1\right)=\left\{y\right\},B_{y}^{S}\left(2\right)=\left\{y,p\right\},B_{y}^{S}\left(3\right)=\left\{x,y,p\right\},B_{y}^{S}\left(4\right)=\left\{x,y,p,q\right\}.$ Similarly, $min_{p}^{S}\left(2\right)=\sqrt{5},min_{p}^{S}\left(3\right)=\sqrt{10},$ $min_{p}^{S}\left(4\right)=5$; $min_{q}^{S}\left(2\right)=\sqrt{5},min_{q}^{S}\left(3\right)=5,min_{q}^{S}\left(4\right)=\sqrt{34}$.*

Suppose the animal radial level is *k* and, at the foraging step i−1, the animal is in the food position $x_{i-1}$, then its next optimal food position for him to forage via LMRFT is

$$x_{i}=\underset{e\in S_{i-1}}{\mathrm{argmin}}\left\{min_{e}^{S_{i-1}}\left(k\right):e\in S_{i-1}\right\},$$

and $S_{i+1}$ is updated by $S_{i}=S_{i-1}-\left\{x_{i}\right\}$. For the initial condition, at the intelligence level *k*, $x_{1}=\underset{e\in S_{0}}{\mathrm{argmin}}\left\{min_{e}^{S_{0}}\left(k\right):e\in S_{0}\right\}$, where $S_{0}$ is the food domain before animal embarks on foraging.

**Example** **2.** *Let us continue Example 1. Let $S_{0}=S$. Take the foraging radial level at k=3. Then, $x_{1}=\mathrm{argmin}\left\{min_{x}^{S_{0}}\left(3\right)=\sqrt{10},min_{y}^{S_{0}}\left(3\right)=\sqrt{13},min_{p}^{S_{0}}\left(3\right)=\sqrt{10},min_{q}^{S_{0}}\left(3\right)=5\right\}=x.$ Indeed, there are two possible solutions: x and p. For simplicity, we simply pick up x. $S_{1}=S_{0}-\left\{x\right\}=\left\{y,p,q\right\}$. $x_{2}=\underset{e\in S_{1}}{\mathrm{argmin}}\left\{min_{e}^{S_{1}}\left(k\right):e\in S_{1}\right\}=argmin\left\{min_{y}^{S_{1}}\left(3\right)=\sqrt{34},min_{p}^{S_{1}}\left(3\right)=5,min_{q}^{S_{1}}\left(3\right)=\sqrt{34}\right\}=p$. Hence, the modelled LMRFT-based optimal path is x to p.*

Similarly, for SMRFT, the iterative steps are as follows

$$x_{i}=\underset{e\in S_{i-1}}{\mathrm{argmin}}\left\{min_{e}^{S_{i-1}}\left(k\right):e\in B_{x_{i-1}}^{S_{i-1}}\left(k\right)\right\},$$

and $S_{i+1}$ is updated by $S_{i}=S_{i-1}-\left\{x_{i}\right\}$.

For the initial condition, at the radial level *k*, $x_{1}$ is set to be the empirical beginning foraging point.

**Example** **3.** *Let us continue Example 1. Let $S_{0}=S$. Take the radial level k=3 again. Assume the initial foraging point for the animal is q. Then $x_{1}=q.$ $B_{x_{1}}^{S_{1}}=B_{q}^{S_{1}}=\left\{x,y,p\right\}$. $x_{2}=\underset{e\in S_{1}}{\mathrm{argmin}}\left\{min_{e}^{S_{1}}\left(k\right):e\in B_{q}^{S_{1}}\right\}=argmin\left\{min_{x}^{S_{1}}\left(3\right)=\sqrt{13},min_{y}^{S_{1}}\left(3\right)=\sqrt{13},min_{p}^{S_{1}}\left(3\right)=\sqrt{10}\right\}=p$. Hence modelled SMRFT-based optimal path is q to p.*

LMRFT and SMRFT would help us identify the modelled optimal foraging paths for animals. Now we could regard these modelled paths as the benchmarks.

Now we need a way link the hypothetically real foraging paths from the experimental results with the benchmarked optimal paths. We adopt a structural metric which reveals the difference among structures. This metric could reveal their spatial properties very well. In our case, it suits our measuring purposes.

- **Introduction of structural metric**

Let strictly ascending vectors $\vec{v}=\left(v_{1},v_{2},\cdots ,v_{m}\right)\in R^{m}$ and $\vec{w}=\left(w_{1},w_{2},\cdots ,w_{n}\right)\in R^{n}$ be arbitrary, where $v_{i}<v_{i+1}$ for all 1≤i≤m−1 and $w_{j}<w_{j+1}$ for all 1≤j≤n−1. We define their common parts (or cp) by a set

$$cp=\{q\in \left\{v_{1},v_{2},\cdots ,v_{m}\right\}\cup \left\{w_{1},w_{2},\cdots ,w_{n}\right\}:max\left\{v_{1},w_{1}\right\}\leq q\leq min\left\{v_{m},w_{n}\right\}\},$$

which is then sorted and characterised by a strictly ascending vector $\vec{z}=\left(z_{1},z_{2},\cdots ,z_{\left|cp\right|}\right)$. Now, the distance between $\vec{v}$ and $\vec{w}$ is defined by

$$\frac{1}{2}\cdot \left[\sqrt{\sum_{i=1}^{m-1}{\left(v_{i+1}-v_{i}\right)}^{2}}+\sqrt{\sum_{j=1}^{n-1}{\left(w_{j+1}-w_{j}\right)}^{2}}\right]-\sqrt{\sum_{k=1}^{|cp|-1}{\left(z_{k+1}-z_{k}\right)}^{2}}.$$

It is shown that this metric satisfies all the axioms for a metric Chen (2022). Unlike the Euclidean metric, this metric studies the difference between the internal structures of the two data points (vectors), and the dimensions for the vector are allowed to be incompatible.

**Example** **4.** *Suppose $\vec{v}=\left(1,4,9,10\right)$ and $\vec{w}=\left(2,3,7,9,12,14,17\right)$. Then, cp={2,4,9,10,3,7} and its representation $\vec{z}=\left(2,3,4,7,9,10\right)$. Hence, the distance between $\vec{v}$ and $\vec{w}$ is computed by $\frac{1}{2}\cdot \left[\sqrt{{\left(4-1\right)}^{2}+{\left(9-4\right)}^{2}+{\left(10-9\right)}^{2}}+\;\sqrt{{\left(3-2\right)}^{2}+{\left(7-3\right)}^{2}+{\left(9-7\right)}^{2}+{\left(12-9\right)}^{2}+{\left(14-12\right)}^{2}+{\left(17-14\right)}^{2}}\right]-\sqrt{{\left(3-2\right)}^{2}+{\left(4-3\right)}^{2}+{\left(7-4\right)}^{2}+{\left(9-7\right)}^{2}+{\left(10-9\right)}^{2}}=2.236759.$*

## 3. Implementation Procedures

In order to extend the flexibility of the modelling, we apply two concepts of locations: a physical one and an intangible one.

- For the physical one, it is simply the measurement between foods, which could be unified or distinct (for controlling and comparative purposes, it is better to unify the food);
- For the intangible locations, it is equivalent to different types or content of foods—each of which retains some features that could be quantified (for example, nutrition, edibility, richness of protein, etc.). The locations of the foods are set according to the 2D location randomly generated via bivariate normal distribution.

We explore LMRFT-based and SRMFT-based optimal foraging strategies in this study. We mainly execute the concept of physical location (two-dimensional location) with a few descriptors regarding the intangible locations. However, one could imitate the same procedures to conduct the concept of intangible location, which shall involve a higher dimension.

- **Measuring procedures for LMRFT-based strategies**

The whole experimental designs regarding extracting the foraging parameters occur as follows:

1. Decide the experimental number of foraging animals and label them, fix the chosen food and decide the number of locations. In our study, we choose three animals: a1 (a male fox, age 5), a2 (a tigress, age 9) and a3 (a male lion, age 7). $\left\{a_{1},a_{2},a_{3}\right\}$ and 20 locations for the experiment.
2. Set up the locations or the features of the food (for example, nutrition and freshness) for the foods. In our study, the locations are generated randomly by a bivariate normal distribution $N∼(\left(0,0\right),\left[\begin{array}{cc} 18 & 10\\ 5 & 9 \end{array}\right])$. The sampled results are shown in Table 1. In the table, we exploit only bivariate normal distribution, which is sufficient for the physical positions. However, if one specifies the locations as intangible (or features of the food) with multiple features, then the simulated data should be generated from some higher-dimensional normal distribution. We track three animals a1–a3. If we specify the location as physical, then the type of chosen foods is some identical fresh meat for the animals. If we specify the location as intangible, then the types of chosen food could be 20 different types of dead prey placed uniformly in the field. No matter which setting we choose, each batch (three batches in total) is presumably represented by a 20-by-2 matrix. Let us use $foodsPos=\{f_{j}^{i}:1\leq j\leq 20\}$ to denote the food positions for $a_{i}$, where 1≤i≤3 (if one considers intangible locations, then $f_{j}^{i}$ is the *j*-th feature vector for animal $a_{i}$). To interpret simplicity, we unify the food in the very beginning. This restriction could be lifted if one studies the intangible locations.
3. Calculate the Euclidean distance matrices for animal 1, animal 2 and animal 3. Let us name the corresponding matrices disMATR 1, disMATR 2 and disMATR 3. The results are presented in Table A1. The table reveals the physical distances between the placed foods at 20 positions. If one measures the intangible distances instead, then the distance matrix shows the feature distances between foods.
4. Rank the distance matrices for the above three distance matrices. The results are shown in Table A2. Let us name the corresponding matrices rank 1, rank 2 and rank 3. The table shows the perceived radii between a given centre (position, or feature) and the other positions (or features) in ranks.
5. Decide the foraging radial levels FRL and foraging steps *N*. In our case, there are four levels for FRL: $fi_{1}=2$, $fi_{2}=3$, $fi_{3}=4$, and $fi_{4}=5$ and N=14 steps. In our demonstrative purpose, we produce the complete results only at level 4 for animal 1. For the results of other levels, regarding other animals, we simply list their results without full explanations. FRL is a set of parameters regarding the animals’ perceived capacity, and N is the searching length for the animals. It should be less than the number of food positions. Based on FRL, we could perform the following recursive steps regarding foraging path mapping:
  - (Step 1) Specify $S_{1}\equiv foods_{1}\left\{1,2,3,\cdots ,20\right\}$. For each foraging radial level $fi_{k}$, for each $x\in foods_{1}$, calculate $min_{x}^{S_{1}}\left(fi_{k}\right)$ and $B_{x}^{S_{1}}\left(fi_{k}\right)$, which are defined in Definitions 2 and 3. The case $fi_{3}=4$ for step 1 is demonstrated at the first column in Table 2. In the table, the left-hand side value indicates the food *x* and the right-hand side value indicates another food position, which is in the 4-th position from *x*, and the value under the arrow indicates the distance between the two food positions. Then, calculate LMRFT-based optimal minimal foraging path. For stage 1; calculate $min\{min_{x}^{S_{1}}\left(fi_{k}\right):x\in S_{1}\}$ and the LMRFT-based optimal target. The results are shown in the last two rows in the same column.
  - (Step 2) Specify $S_{2}\equiv foods_{2}=S_{1}-\left\{\;\mathrm{new}\;\mathrm{eaten}\;\mathrm{target}\right\}$. For each foraging radial level $fi_{k}$, for each $x\in foods_{2}$, calculate $min_{x}^{S_{2}}\left(fi_{k}\right)$ and $B_{x}^{S_{2}}\left(fi_{k}\right)$. Then, calculate the $min\{min_{x}^{S_{2}}\left(fi_{k}\right):x\in S_{2}\}$ and LMRFT-based optimal target. The demonstrative calculation is shown in the second column of Table 2. By repeating the steps, we obtain the general description for the n+1 step from the *n* step.
  - (Step n+1) Specify $S_{n+1}\equiv foods_{n+1}=S_{n}-neweatentarget$. For each foraging radial level $fi_{k}$, and for each $x\in foods_{n+1}$, calculate $min_{x}^{S_{n+1}}\left(fi_{k}\right)$ and $B_{x}^{S_{n+1}}\left(fi_{k}\right)$. Then, calculate $min\{min_{x}^{S_{n+1}}\left(fi_{k}\right):x\in S_{n+1}\}$ and LMRFT-based optimal target. The demonstrative calculations are shown up to n=4 or step 5 as presented in Table 2.
6. Calculate LMRFT-based optimal paths and distances by collecting all the eaten targets in the above steps. The results are presented in Table 3.
7. Record the hypothetically real paths with distances for animals under the provided foods and food positions. In our case, the results are presented in Table 4.
8. Calculate the structural distance between the modelled paths and the hypothetically real paths and find out the most matched paths to yield an optimal foraging radial level. The results are presented in Section 4.5.
9. Compare the foraging strategies between the animals. The results are presented in Section 4.5.

**Remark** **1.** *As for SMRFT, we simply replace the algorithms from step 1 up to step n+1 with the iterative steps formulated in Section 2.*

## 4. Results

In this section, we present the results under LMRFT and SMRFT.

### 4.1. Preliminary Settings

For each animal, i.e., a sample, we generate 20 two-dimensional data points to represent the positions of the foods via a given bivariate normal distribution. For the three animals, the generated data regarding the food positions are listed in Table 1.

### 4.2. Empirical Study

Suppose the hypothetically real foraging paths for the three animals are in agreement with Table 4.

### 4.3. LMRFT Results

Following the implementation procedures described in Section 3, we produce the results as (partially) shown in Table 2. Furthermore, the modelled optimal paths and their distances are presented in Table 3.

### 4.4. SMRFT Results

Following the implementation procedures and Remark 1 described in Section 3, we produce modelled SMRFT-based optimal paths and their distances as, presented in Table 5.

### 4.5. Matched Foraging Strategies

By adopting the structural metric mentioned in Section 2, we calculate the distances between modelled optimal paths and hypothetically real paths under various radial levels. By matching the closest one, in terms of radial levels, we obtain the following results. LMRFT-based optimal distances for animal 1 are 22.917 between animal 1’s hypothetically real path and optimal path at radial level 2; 21.229 between animal 1’s hypothetically real path and optimal path at radial level 3; 22.335 between animal 1’s hypothetically real path and optimal path at radial level 4; and 22.025 between animal 1’s hypothetically real path and optimal path at radial level 5. Hence, LMRFT-based foraging radial level for animal 1 is 3. Moreover, LMRFT-based optimal distances for animal 2 are 21.025, 21.023, 21.993, and 22.655 at radial level 2, 3, 4 and 5, respectively. Hence, LMRFT-based foraging radial level for animal 2 is two. Similarly, LMRFT-based optimal distances for animal 3 are 18.792, 19.23, 18.556, 18.006 at level 2, 3, 4 and 5. Hence, LMRFT-based foraging radial level for animal 3 is five. In the same manner, we calculate SMRFT-based optimal distances for animal 1, 2 and 3 at level 2, 3, 4 and 5: 19.952, 19.48, 19.623, 19.253; 19.443, 18.364, 17.977, 17.765; 16.582, 15.758, 16.411, 16.388, i.e., SMRFT-based foraging strategies for animal 1, 2 and 3 are five, five and two, respectively. Therefore, in a LMRFT-based scale of foraging radial levels, animal 3 is better than animal 1, and animal 1 is better than animal 2; but, in a SMRFT-based scale of radial levels, animal 1 is equivalent to animal 2, and both are better than animal 3.

## 5. Conclusions and Future Work

We come up with a modelling approach for measuring animal foraging strategies: LMRFT-based and SMRFT-based strategies. This modelling extracts some parameters regarding animals’ dynamical foraging behaviours, particularly their foraging paths. Based on different radial levels, we construct their LMRFT-based and SMRFT-based optimal foraging paths. By comparing the hypothetically real foraging paths of animals with the optimal paths, we calculate their structural distances and identify their radial level. This modelling is implemented by a series of experimental designs. There are some characteristics of this modelling and its implementation.

1. It is related to artificial intelligence and machine learning techniques, and thus could be further expanded by these subjects. For example, one could further consider animal grazing time and bite rate.
2. The measurement of foraging radial level is straightforward and comprehensible.
3. There are very few restrictions in terms of applications. In essence, all the parameters regarding the model could be added or removed. This gives us a high degree of freedom in choosing the optimal parameters or methods.
4. The structural metric is adopted to reflect the modelled optimal paths and the (simulated) empirical paths. This metric suits the spatial data and could reveal the difference between structures—in our case, the foraging dynamics.
5. This model provides a general platform for exploring the foraging radial levels. One could easily amend the models to fit their specific purposes and targets.

There are also some limitations and suggestions:

1. The foraging radial level is a composite index, which could be further expanded by other detailed factors or variables; for example, we could take the travel risk for foraging into consideration. In addition, the perceived distances are assumed to be the spatial Euclidean distances. This might not faithfully represent animal foraging cognition regarding geographical distances—other metrics or machine learning techniques could be combined to yield an optimal description Rodríguez-Malagón et al. (2020).
2. The collective foraging radial levels for animals are not considered in this study. If one wants to measure their collective foraging radial levels, a complete dynamical interaction and behavioural models must be considered. Collective foraging involves complex social interactions. A sole concept of radial level would not be enough to capture such interaction. Some other models concerning social behaviours and collective learning should be engaged instead (Evans et al. 2019; Lemanski et al. 2021).
3. An empirical experiment is lacking in this study. One could actually conduct an experiment based on this modelling and its implementation to reach empirical results. For other researchers, if their research tools, such as GPS, are well equipped, then they could conduct a real experiment to extract related parameters for checking our modelling approaches. This could further enrich the modelling or correct some setting of experimental designs Eliezer et al. (2022).

## Figures and Tables

**Table 1:** Experimental designs regarding the setting of placed food 2D positions foodsPos for animal 1 (left block, or ${\left\{f_{j}^{1}\right\}}_{j=1}^{20}$), animal 2 (middle block, or ${\left\{f_{j}^{2}\right\}}_{j=1}^{20}$) and animal 3 (right block, or ${\left\{f_{j}^{3}\right\}}_{j=1}^{20}$).

Label	*X* Axis	*Y* Axis	*X* Axis	*Y* Axis	*X* Axis	*Y* Axis
$f_{1}^{i}$	5.144	5.100	2.497	3.872	−3.991	−2.857
$f_{2}^{i}$	3.599	5.875	−5.704	1.842	−3.353	1.956
$f_{3}^{i}$	−3.827	−3.001	−2.490	3.208	−3.834	−2.211
$f_{4}^{i}$	0.523	1.476	−0.116	−0.715	−8.300	−4.745
$f_{5}^{i}$	−2.426	1.196	−5.141	−2.414	−1.608	0.831
$f_{6}^{i}$	2.964	3.077	2.448	0.911	1.692	−1.398
$f_{7}^{i}$	1.206	−0.038	−3.359	4.763	3.451	0.995
$f_{8}^{i}$	0.810	1.658	−3.880	0.099	0.298	4.509
$f_{9}^{i}$	2.517	−3.601	−7.157	−1.276	−1.484	3.148
$f_{10}^{i}$	−6.446	−3.706	2.141	0.392	12.134	−1.437
$f_{11}^{i}$	2.640	−0.369	3.361	2.267	1.058	−2.188
$f_{12}^{i}$	11.790	−1.516	2.280	−2.018	−6.652	−0.437
$f_{13}^{i}$	6.280	1.790	1.010	4.840	−4.290	−0.811
$f_{14}^{i}$	2.901	2.826	−6.651	−1.185	−0.353	0.198
$f_{15}^{i}$	−2.970	−5.068	2.314	−1.310	2.642	0.310
$f_{16}^{i}$	1.808	−1.380	2.030	3.666	−0.634	0.266
$f_{17}^{i}$	2.880	1.492	3.005	−1.613	6.764	1.434
$f_{18}^{i}$	0.831	−2.256	1.953	1.340	3.349	−1.626
$f_{19}^{i}$	−6.048	−3.446	0.970	4.742	2.779	3.590
$f_{20}^{i}$	−1.168	−0.798	−7.194	−4.894	−0.690	−4.584

**Table 2:** Partial computation (5 steps) for LMRFT-based optimal foraging path among 14 steps for animal 1.

Step 1	Step 2	Step 3	Step 4	Step 5
$1\overset{4}{\underset{3.1942}{\to }}14$	$1\overset{4}{\underset{3.1942}{\to }}14$	$1\overset{4}{\underset{3.1942}{\to }}13$	$1\overset{4}{\underset{3.1942}{\to }}13$	$1\overset{4}{\underset{3.1942}{\to }}12$
$2\overset{4}{\underset{3.1275}{\to }}14$	$2\overset{4}{\underset{3.1275}{\to }}14$	$2\overset{4}{\underset{3.1275}{\to }}13$	$2\overset{4}{\underset{3.1275}{\to }}13$	$2\overset{4}{\underset{3.1275}{\to }}12$
$3\overset{4}{\underset{2.7125}{\to }}10$	$3\overset{4}{\underset{2.7125}{\to }}10$	$3\overset{4}{\underset{2.7125}{\to }}9$	$3\overset{4}{\underset{2.7125}{\to }}9$	$3\overset{4}{\underset{2.7125}{\to }}8$
$4\overset{4}{\underset{2.3575}{\to }}17$	$4\overset{4}{\underset{2.3575}{\to }}16$	$4\overset{4}{\underset{2.7349}{\to }}13$	$4\overset{4}{\underset{2.808}{\to }}10$	$4\overset{4}{\underset{2.8329}{\to }}16$
$5\overset{4}{\underset{3.2688}{\to }}8$	$5\overset{4}{\underset{3.2688}{\to }}8$	$5\overset{4}{\underset{3.2688}{\to }}7$	$5\overset{4}{\underset{3.2688}{\to }}7$	$5\overset{4}{\underset{4.4245}{\to }}3$
$6\overset{4}{\underset{2.5793}{\to }}8$	$6\overset{4}{\underset{2.5793}{\to }}8$	$6\overset{4}{\underset{2.5793}{\to }}7$	$6\overset{4}{\underset{2.8686}{\to }}2$	$6\overset{4}{\underset{2.9198}{\to }}4$
$7\overset{4}{\underset{1.6609}{\to }}4$	$7\overset{4}{\underset{1.7417}{\to }}8$	7 is eaten	7 was eaten	7 was eaten
$8\overset{4}{\underset{2.0765}{\to }}17$	$8\overset{4}{\underset{2.0765}{\to }}16$	$8\overset{4}{\underset{2.3948}{\to }}13$	$8\overset{4}{\underset{2.5793}{\to }}6$	8 is eaten
$9\overset{4}{\underset{3.2347}{\to }}11$	$9\overset{4}{\underset{3.7965}{\to }}7$	$9\overset{4}{\underset{4.63}{\to }}18$	$9\overset{4}{\underset{4.63}{\to }}17$	$9\overset{4}{\underset{4.63}{\to }}16$
$10\overset{4}{\underset{3.7331}{\to }}15$	$10\overset{4}{\underset{3.7331}{\to }}15$	$10\overset{4}{\underset{3.7331}{\to }}14$	$10\overset{4}{\underset{3.7331}{\to }}14$	$10\overset{4}{\underset{3.7331}{\to }}13$
$11\overset{4}{\underset{1.8766}{\to }}17$	$11\overset{4}{\underset{2.6138}{\to }}17$	$11\overset{4}{\underset{2.7304}{\to }}7$	$11\overset{4}{\underset{2.808}{\to }}4$	$11\overset{4}{\underset{3.2053}{\to }}12$
$12\overset{4}{\underset{9.3783}{\to }}1$	$12\overset{4}{\underset{9.3783}{\to }}1$	$12\overset{4}{\underset{9.3783}{\to }}1$	$12\overset{4}{\underset{9.3783}{\to }}1$	$12\overset{4}{\underset{9.3783}{\to }}1$
$13\overset{4}{\underset{3.5337}{\to }}14$	$13\overset{4}{\underset{3.5337}{\to }}14$	$13\overset{4}{\underset{3.5337}{\to }}13$	$13\overset{4}{\underset{3.5563}{\to }}6$	$13\overset{4}{\underset{3.5563}{\to }}6$
$14\overset{4}{\underset{2.3948}{\to }}8$	$14\overset{4}{\underset{2.3948}{\to }}8$	$14\overset{4}{\underset{2.3948}{\to }}7$	$14\overset{4}{\underset{2.7349}{\to }}4$	$14\overset{4}{\underset{3.1275}{\to }}2$
$15\overset{4}{\underset{3.7331}{\to }}10$	$15\overset{4}{\underset{3.7331}{\to }}10$	$15\overset{4}{\underset{3.7331}{\to }}9$	$15\overset{4}{\underset{3.7331}{\to }}9$	$15\overset{4}{\underset{3.7331}{\to }}8$
$16\overset{4}{\underset{1.4707}{\to }}7$	16 is eaten	16 was eaten	16 was eaten	16 was eaten
$17\overset{4}{\underset{1.8766}{\to }}11$	$17\overset{4}{\underset{1.8766}{\to }}11$	$17\overset{4}{\underset{1.8766}{\to }}10$	17 is eaten	17 was eaten
$18\overset{4}{\underset{2.2488}{\to }}7$	$18\overset{4}{\underset{2.474}{\to }}19$	$18\overset{4}{\underset{2.6138}{\to }}10$	$18\overset{4}{\underset{2.6138}{\to }}10$	$18\overset{4}{\underset{2.6138}{\to }}9$
$19\overset{4}{\underset{3.4794}{\to }}15$	$19\overset{4}{\underset{3.4794}{\to }}15$	$19\overset{4}{\underset{3.4794}{\to }}14$	$19\overset{4}{\underset{3.4794}{\to }}14$	$19\overset{4}{\underset{3.4794}{\to }}13$
$20\overset{4}{\underset{2.4921}{\to }}7$	$20\overset{4}{\underset{2.4921}{\to }}7$	$20\overset{4}{\underset{2.8329}{\to }}4$	$20\overset{4}{\underset{2.8329}{\to }}4$	$20\overset{4}{\underset{2.8329}{\to }}4$
$16\overset{4}{\underset{1.4707}{\to }}7$	$7\overset{4}{\underset{1.7417}{\to }}8$	$17\overset{4}{\underset{1.8766}{\to }}10$	$8\overset{4}{\underset{2.5793}{\to }}6$	$18\overset{4}{\underset{2.6138}{\to }}9$
target: 16	target: 7	target: 17	target: 8	target: 18

**Table 3:** LMRFT-based optimal paths and distances for animal 1, 2 and 3 under foraging radial levels 2, 3, 4 and 5.

Cases	LMRFT-Based Optimal Paths and Distances
$a_{1}$, fi=2	$6\overset{2}{\underset{2.9198}{\to }}4\overset{2}{\underset{8.6838}{\to }}10\overset{2}{\underset{9.6792}{\to }}11\overset{2}{\underset{1.3095}{\to }}16\overset{2}{\underset{4.3456}{\to }}14\overset{2}{\underset{3.1942}{\to }}1\overset{2}{\underset{6.4739}{\to }}$
	$7\overset{2}{\underset{1.7417}{\to }}8\overset{2}{\underset{5.5291}{\to }}9\overset{2}{\underset{6.3722}{\to }}3\overset{2}{\underset{4.4245}{\to }}5\overset{2}{\underset{4.7460}{\to }}18\overset{2}{\underset{6.7863}{\to }}13$
$a_{1}$, fi=3	$16\overset{3}{\underset{4.3456}{\to }}14\overset{3}{\underset{2.7349}{\to }}4\overset{3}{\underset{1.6609}{\to }}7\overset{3}{\underset{2.2685}{\to }}17\overset{3}{\underset{8.0726}{\to }}3\overset{3}{\underset{4.7170}{\to }}18\overset{3}{\underset{3.9136}{\to }}8$
	$\overset{3}{\underset{5.0552}{\to }}2\overset{3}{\underset{2.8686}{\to }}6\overset{3}{\underset{11.1254}{\to }}19\overset{3}{\underset{9.2171}{\to }}11\overset{3}{\underset{3.8317}{\to }}20\overset{3}{\underset{4.6346}{\to }}15$
$a_{1}$, fi=4	$16\overset{4}{\underset{1.47074}{\to }}7\overset{4}{\underset{2.26848}{\to }}17\overset{4}{\underset{2.07648}{\to }}8\overset{4}{\underset{3.91359}{\to }}18\overset{4}{\underset{4.71699}{\to }}3\overset{4}{\underset{6.24118}{\to }}4\overset{4}{\underset{2.91982}{\to }}$
	$6\overset{4}{\underset{0.25901}{\to }}14\overset{4}{\underset{3.20529}{\to }}11\overset{4}{\underset{7.31765}{\to }}15\overset{4}{\underset{4.63455}{\to }}20\overset{4}{\underset{7.88401}{\to }}13\overset{4}{\underset{8.72556}{\to }}5$
$a_{1}$, fi=5	$7\overset{5}{\underset{2.26848}{\to }}17\overset{5}{\underset{4.27153}{\to }}18\overset{5}{\underset{3.91359}{\to }}8\overset{5}{\underset{0.34052}{\to }}4\overset{5}{\underset{2.73486}{\to }}14\overset{5}{\underset{8.89986}{\to }}3\overset{5}{\underset{9.11327}{\to }}$
	$6\overset{5}{\underset{3.46105}{\to }}11\overset{5}{\underset{3.83169}{\to }}20\overset{5}{\underset{4.63455}{\to }}15\overset{5}{\underset{11.51430}{\to }}13\overset{5}{\underset{8.72556}{\to }}5\overset{5}{\underset{4.95622}{\to }}16$
$a_{2}$, fi=2	$13\overset{2}{\underset{1.77426}{\to }}1\overset{2}{\underset{10.94087}{\to }}9\overset{2}{\underset{9.85110}{\to }}6\overset{2}{\underset{2.93366}{\to }}12\overset{2}{\underset{0.70807}{\to }}15\overset{2}{\underset{1.71078}{\to }}10\overset{2}{\underset{3.27600}{\to }}$
	$16\overset{2}{\underset{1.93023}{\to }}11\overset{2}{\underset{5.92592}{\to }}3\overset{2}{\underset{6.21583}{\to }}5\overset{2}{\underset{4.29356}{\to }}2\overset{2}{\underset{6.14527}{\to }}4\overset{2}{\underset{3.85133}{\to }}8$
$a_{2}$, fi=3	$6\overset{3}{\underset{2.2256}{\to }}15\overset{3}{\underset{4.9842}{\to }}16\overset{3}{\underset{2.3270}{\to }}18\overset{3}{\underset{3.5406}{\to }}19\overset{3}{\underset{1.7570}{\to }}1\overset{3}{\underset{10.4521}{\to }}$
	$14\overset{3}{\underset{9.6648}{\to }}17\overset{3}{\underset{2.1824}{\to }}10\overset{3}{\underset{7.8042}{\to }}5\overset{3}{\underset{6.2158}{\to }}3\overset{3}{\underset{3.4920}{\to }}2\overset{3}{\underset{3.4402}{\to }}9\overset{3}{\underset{7.0637}{\to }}4$
$a_{2}$, fi=4	$16\overset{4}{\underset{2.7860}{\to }}6\overset{4}{\underset{2.2256}{\to }}15\overset{4}{\underset{5.1853}{\to }}1\overset{4}{\underset{3.4982}{\to }}10\overset{4}{\underset{7.8042}{\to }}5\overset{4}{\underset{8.0261}{\to }}18\overset{4}{\underset{8.9663}{\to }}$
	$14\overset{4}{\underset{3.1718}{\to }}2\overset{4}{\underset{3.4920}{\to }}3\overset{4}{\underset{4.5852}{\to }}4\overset{4}{\underset{4.5804}{\to }}11\overset{4}{\underset{7.1686}{\to }}7\overset{4}{\underset{4.6934}{\to }}8$
$a_{2}$, fi=5	$1\overset{5}{\underset{3.4982}{\to }}10\overset{5}{\underset{3.2760}{\to }}16\overset{5}{\underset{4.9842}{\to }}15\overset{5}{\underset{2.2256}{\to }}6\overset{5}{\underset{9.3368}{\to }}14\overset{5}{\underset{8.9663}{\to }}18\overset{5}{\underset{5.9637}{\to }}$
	$8\overset{5}{\underset{3.4055}{\to }}3\overset{5}{\underset{5.9259}{\to }}11\overset{5}{\underset{9.7056}{\to }}5\overset{5}{\underset{5.3048}{\to }}4\overset{5}{\underset{5.5634}{\to }}19\overset{5}{\underset{7.2770}{\to }}2$
$a_{3}$, fi=2	$14\overset{2}{\underset{4.7506}{\to }}1\overset{2}{\underset{5.8679}{\to }}6\overset{2}{\underset{2.9698}{\to }}7\overset{2}{\underset{5.0616}{\to }}5\overset{2}{\underset{3.7694}{\to }}3\overset{2}{\underset{6.9495}{\to }}15\overset{2}{\underset{6.2168}{\to }}$
	$2\overset{2}{\underset{4.4548}{\to }}8\overset{2}{\underset{6.7397}{\to }}11\overset{2}{\underset{7.9063}{\to }}12\overset{2}{\underset{6.2901}{\to }}9\overset{2}{\underset{4.8527}{\to }}13\overset{2}{\underset{3.8112}{\to }}16$
$a_{3}$, fi=3	$16\overset{3}{\underset{4.0466}{\to }}3\overset{3}{\underset{5.5862}{\to }}6\overset{3}{\underset{1.9544}{\to }}15\overset{3}{\underset{4.2818}{\to }}5\overset{3}{\underset{2.3203}{\to }}9\overset{3}{\underset{4.8527}{\to }}13\overset{3}{\underset{7.6825}{\to }}$
	$18\overset{3}{\underset{5.2476}{\to }}19\overset{3}{\underset{6.0283}{\to }}11\overset{3}{\underset{5.0937}{\to }}1\overset{3}{\underset{8.3799}{\to }}7\overset{3}{\underset{6.8713}{\to }}2\overset{3}{\underset{3.4772}{\to }}14$
$a_{3}$, fi=4	$6\overset{4}{\underset{3.9831}{\to }}5\overset{4}{\underset{3.1446}{\to }}13\overset{4}{\underset{7.6825}{\to }}18\overset{4}{\underset{2.3585}{\to }}11\overset{4}{\underset{5.9103}{\to }}9\overset{4}{\underset{5.0075}{\to }}15\overset{4}{\underset{7.3504}{\to }}$
	$1\overset{4}{\underset{8.3799}{\to }}7\overset{4}{\underset{7.9593}{\to }}3\overset{4}{\underset{4.1946}{\to }}2\overset{4}{\underset{4.4548}{\to }}8\overset{4}{\underset{2.6456}{\to }}19\overset{4}{\underset{4.7641}{\to }}16$
$a_{3}$, fi=5	$5\overset{5}{\underset{3.98307}{\to }}6\overset{5}{\underset{6.01117}{\to }}13\overset{5}{\underset{5.52262}{\to }}11\overset{5}{\underset{5.91029}{\to }}9\overset{5}{\underset{5.00748}{\to }}15\overset{5}{\underset{1.05996}{\to }}7\overset{5}{\underset{7.95925}{\to }}$
	$3\overset{5}{\underset{4.23315}{\to }}14\overset{5}{\underset{0.28896}{\to }}16\overset{5}{\underset{4.58521}{\to }}1\overset{5}{\underset{9.34870}{\to }}19\overset{5}{\underset{6.34558}{\to }}2\overset{5}{\underset{7.59968}{\to }}18$

**Table 4:** The hypothetically real foraging paths for animal 1, 2 and 3. These results, which are based on simulated data, represent the real foraging paths for each animal.

Animals	Hypothetically Real Paths
$a_{1}$	$7\underset{5.3838}{\to }9\underset{14.3692}{\to }10\underset{18.8127}{\to }12\underset{13.5455}{\to }17\underset{8.3937}{\to }5\underset{4.3913}{\to }1\underset{9.3487}{\to }$
	$19\underset{2.6456}{\to }8\underset{4.3443}{\to }16\underset{4.4099}{\to }18\underset{7.6825}{\to }13\underset{2.9215}{\to }2\underset{6.0589}{\to }6$
$a_{2}$	$11\underset{6.0524}{\to }2\underset{15.8547}{\to }10\underset{18.8127}{\to }12\underset{10.2036}{\to }7\underset{2.9698}{\to }6\underset{3.9776}{\to }20\underset{8.8792}{\to }$
	$19\underset{5.2476}{\to }18\underset{4.4099}{\to }16\underset{4.3443}{\to }8\underset{8.5240}{\to }1\underset{2.0680}{\to }13\underset{1.4720}{\to }3$
$a_{3}$	$6\underset{8.3994}{\to }12\underset{3.5964}{\to }1\underset{4.8556}{\to }2\underset{10.1304}{\to }17\underset{4.2725}{\to }15\underset{9.6519}{\to }10\underset{14.3692}{\to }$
	$9\underset{5.9103}{\to }11\underset{2.9805}{\to }16\underset{1.1263}{\to }5\underset{5.1823}{\to }19\underset{8.8792}{\to }20\underset{7.6126}{\to }4$

**Table 5:** SMRFT-based optimal paths and distances for animal 1, 2 and 3 under foraging radial levels 2, 3, 4 and 5.

Cases	SMRFT-Based Optimal Paths and Distances
$a_{1}$, fi=2	$7\overset{2}{\underset{1.3118}{\to }}16\overset{2}{\underset{2.6138}{\to }}11\overset{2}{\underset{1.5869}{\to }}17\overset{2}{\underset{2.3948}{\to }}14\overset{2}{\underset{2.8686}{\to }}6\overset{2}{\underset{3.1532}{\to }}8\overset{2}{\underset{2.9617}{\to }}$
	$4\overset{2}{\underset{2.4740}{\to }}20\overset{2}{\underset{4.7460}{\to }}5\overset{2}{\underset{2.2659}{\to }}3\overset{2}{\underset{3.7331}{\to }}15\overset{2}{\underset{6.9817}{\to }}19\overset{2}{\underset{8.9636}{\to }}10$
$a_{1}$, fi=3	$7\overset{3}{\underset{1.3118}{\to }}16\overset{3}{\underset{2.4740}{\to }}18\overset{3}{\underset{2.8329}{\to }}20\overset{3}{\underset{2.3575}{\to }}4\overset{3}{\underset{1.5869}{\to }}17\overset{3}{\underset{2.3948}{\to }}14\overset{3}{\underset{2.7304}{\to }}$
	$8\overset{3}{\underset{2.9739}{\to }}6\overset{3}{\underset{3.4996}{\to }}1\overset{3}{\underset{4.8856}{\to }}13\overset{3}{\underset{5.3021}{\to }}11\overset{3}{\underset{5.8885}{\to }}5\overset{3}{\underset{2.2659}{\to }}3$
$a_{1}$, fi=4	$7\overset{4}{\underset{1.8766}{\to }}11\overset{4}{\underset{1.5869}{\to }}17\overset{4}{\underset{2.3948}{\to }}8\overset{4}{\underset{2.8329}{\to }}4\overset{4}{\underset{2.8686}{\to }}6\overset{4}{\underset{3.1275}{\to }}2\overset{4}{\underset{3.4996}{\to }}$
	$1\overset{4}{\underset{4.3456}{\to }}14\overset{4}{\underset{2.3315}{\to }}16\overset{4}{\underset{2.4740}{\to }}18\overset{4}{\underset{2.2659}{\to }}3\overset{4}{\underset{3.7331}{\to }}10\overset{4}{\underset{4.6346}{\to }}15$
$a_{1}$, fi=5	$7\overset{5}{\underset{2.0765}{\to }}8\overset{5}{\underset{1.5869}{\to }}6\overset{5}{\underset{2.7349}{\to }}4\overset{5}{\underset{1.8766}{\to }}11\overset{5}{\underset{2.3315}{\to }}16\overset{5}{\underset{3.4124}{\to }}17\overset{5}{\underset{3.1942}{\to }}$
	$1\overset{5}{\underset{4.4245}{\to }}5\overset{5}{\underset{2.2659}{\to }}3\overset{5}{\underset{3.7331}{\to }}10\overset{5}{\underset{2.4740}{\to }}18\overset{5}{\underset{3.5337}{\to }}14\overset{5}{\underset{5.6796}{\to }}9$
$a_{2}$, fi=2	$11\overset{2}{\underset{0.65501}{\to }}6\overset{2}{\underset{1.71078}{\to }}10\overset{2}{\underset{2.58941}{\to }}18\overset{2}{\underset{1.51006}{\to }}16\overset{2}{\underset{1.77426}{\to }}1\overset{2}{\underset{3.78524}{\to }}19\overset{2}{\underset{4.37011}{\to }}$
	$13\overset{2}{\underset{3.40552}{\to }}3\overset{2}{\underset{4.69337}{\to }}7\overset{2}{\underset{3.17182}{\to }}2\overset{2}{\underset{3.05339}{\to }}8\overset{2}{\underset{2.31541}{\to }}5\overset{2}{\underset{3.74879}{\to }}14$
$a_{2}$, fi=3	$11\overset{3}{\underset{0.65501}{\to }}6\overset{3}{\underset{1.71078}{\to }}10\overset{3}{\underset{0.75405}{\to }}15\overset{3}{\underset{2.72764}{\to }}12\overset{3}{\underset{3.24737}{\to }}4\overset{3}{\underset{2.58941}{\to }}18\overset{3}{\underset{1.51006}{\to }}$
	$16\overset{3}{\underset{1.75697}{\to }}19\overset{3}{\underset{3.86193}{\to }}13\overset{3}{\underset{3.40552}{\to }}3\overset{3}{\underset{2.81183}{\to }}8\overset{3}{\underset{2.31541}{\to }}5\overset{3}{\underset{3.17182}{\to }}14$
$a_{2}$, fi=4	$11\overset{4}{\underset{0.65501}{\to }}6\overset{4}{\underset{0.75405}{\to }}15\overset{4}{\underset{2.18240}{\to }}10\overset{4}{\underset{2.72764}{\to }}12\overset{4}{\underset{3.24737}{\to }}4\overset{4}{\underset{2.81183}{\to }}8\overset{4}{\underset{2.31541}{\to }}$
	$5\overset{4}{\underset{3.17182}{\to }}14\overset{4}{\underset{3.49198}{\to }}2\overset{4}{\underset{3.78524}{\to }}3\overset{4}{\underset{1.55523}{\to }}13\overset{4}{\underset{1.51006}{\to }}16\overset{4}{\underset{2.58941}{\to }}1$
$a_{2}$, fi=5	$11\overset{5}{\underset{0.65501}{\to }}6\overset{5}{\underset{0.75405}{\to }}15\overset{5}{\underset{2.18240}{\to }}10\overset{5}{\underset{2.72764}{\to }}12\overset{5}{\underset{1.51006}{\to }}16\overset{5}{\underset{1.77426}{\to }}1\overset{5}{\underset{3.40552}{\to }}$
	$3\overset{5}{\underset{3.17182}{\to }}2\overset{5}{\underset{3.05339}{\to }}8\overset{5}{\underset{2.31541}{\to }}5\overset{5}{\underset{3.24737}{\to }}4\overset{5}{\underset{3.62446}{\to }}13\overset{5}{\underset{6.31969}{\to }}7$
$a_{3}$, fi=2	$6\overset{2}{\underset{2.7717}{\to }}11\overset{2}{\underset{2.6235}{\to }}18\overset{2}{\underset{2.9971}{\to }}15\overset{2}{\underset{3.3421}{\to }}7\overset{2}{\underset{4.2852}{\to }}19\overset{2}{\underset{4.1424}{\to }}8\overset{2}{\underset{2.3203}{\to }}$
	$9\overset{2}{\underset{2.9215}{\to }}2\overset{2}{\underset{1.4057}{\to }}5\overset{2}{\underset{3.8112}{\to }}16\overset{2}{\underset{4.2332}{\to }}14\overset{2}{\underset{2.0680}{\to }}13\overset{2}{\underset{3.3289}{\to }}3$
$a_{3}$, fi=3	$6\overset{3}{\underset{2.3585}{\to }}18\overset{3}{\underset{2.9573}{\to }}11\overset{3}{\underset{1.4057}{\to }}14\overset{3}{\underset{2.0761}{\to }}5\overset{3}{\underset{2.9215}{\to }}2\overset{3}{\underset{2.0680}{\to }}13\overset{3}{\underset{3.3289}{\to }}$
	$3\overset{3}{\underset{3.7259}{\to }}1\overset{3}{\underset{5.9201}{\to }}20\overset{3}{\underset{3.2762}{\to }}15\overset{3}{\underset{3.3421}{\to }}7\overset{3}{\underset{4.2852}{\to }}19\overset{3}{\underset{3.0049}{\to }}9$
$a_{3}$, fi=4	$6\overset{4}{\underset{2.0617}{\to }}15\overset{4}{\underset{2.6807}{\to }}7\overset{4}{\underset{4.1274}{\to }}18\overset{4}{\underset{1.4057}{\to }}14\overset{4}{\underset{2.0761}{\to }}5\overset{4}{\underset{2.2419}{\to }}9\overset{4}{\underset{3.2016}{\to }}$
	$2\overset{4}{\underset{2.0680}{\to }}13\overset{4}{\underset{3.3289}{\to }}3\overset{4}{\underset{3.7259}{\to }}1\overset{4}{\underset{4.3443}{\to }}16\overset{4}{\underset{6.0283}{\to }}11\overset{4}{\underset{4.5313}{\to }}19$
$a_{3}$, fi=5	$6\overset{5}{\underset{1.4057}{\to }}14\overset{5}{\underset{2.0761}{\to }}5\overset{5}{\underset{2.2419}{\to }}9\overset{5}{\underset{3.2016}{\to }}2\overset{5}{\underset{1.4720}{\to }}3\overset{5}{\underset{3.5964}{\to }}1\overset{5}{\underset{3.8112}{\to }}$
	$13\overset{5}{\underset{2.9573}{\to }}11\overset{5}{\underset{2.0617}{\to }}15\overset{5}{\underset{2.6807}{\to }}7\overset{5}{\underset{4.4099}{\to }}16\overset{5}{\underset{5.0057}{\to }}18\overset{5}{\underset{6.0895}{\to }}17$

## Data Availability

The author confirm that the data supporting the findings of this study are available within the article.

## Appendix A. Distance Matrices

**Table A1:** Euclidean distance matrices for animal 1 (top block), animal 2 (middle block) and animal 3 (bottom block).

	$f_{1}^{1}$	$f_{2}^{1}$	$f_{3}^{1}$	⋯	$f_{18}^{1}$	$f_{19}^{1}$	$f_{20}^{1}$
$f_{1}^{1}$	0	1.7282	12.0868	⋯	8.5269	14.082	8.6383
$f_{2}^{1}$	1.7282	0	11.5716	⋯	8.5883	13.414	8.1998
$f_{3}^{1}$	12.0868	11.5716	0	⋯	4.717	2.2659	3.4529
⋮	⋮	⋮	⋮	⋮	⋮	⋮	⋮
$f_{18}^{1}$	8.5269	8.5883	4.717	⋯	0	6.9817	2.474
$f_{19}^{1}$	14.082	13.414	2.2659	⋯	6.9817	0	5.5529
$f_{20}^{1}$	8.6383	8.1998	3.4529	⋯	2.474	5.5529	0
	$f_{1}^{2}$	$f_{2}^{2}$	$f_{3}^{2}$	⋯	$f_{18}^{2}$	$f_{19}^{2}$	$f_{20}^{2}$
$f_{1}^{2}$	0	8.4483	5.031	⋯	2.5894	1.757	13.0677
$f_{2}^{2}$	8.4483	0	3.492	⋯	7.6733	7.277	6.8993
$f_{3}^{2}$	5.031	3.492	0	⋯	4.8196	3.7852	9.3688
⋮	⋮	⋮	⋮	⋮	⋮	⋮	⋮
$f_{18}^{2}$	2.5894	7.6733	4.8196	⋯	0	3.5406	11.0697
$f_{19}^{2}$	1.757	7.277	3.7852	⋯	3.5406	0	12.6299
$f_{20}^{2}$	13.0677	6.8993	9.3688	⋯	11.0697	12.6299	0
	$f_{1}^{3}$	$f_{2}^{3}$	$f_{3}^{3}$	⋯	$f_{18}^{3}$	$f_{19}^{3}$	$f_{20}^{3}$
$f_{1}^{3}$	0	4.8556	0.66538	⋯	7.4428	9.3487	3.7259
$f_{2}^{3}$	4.85562	0	4.19457	⋯	7.5997	6.3456	7.0616
$f_{3}^{3}$	0.66538	4.1946	0	⋯	7.2073	8.7966	3.9399
⋮	⋮	⋮	⋮	⋮	⋮	⋮	⋮
$f_{18}^{3}$	7.44283	7.5997	7.2073	⋯	0	5.2476	5.0057
$f_{19}^{3}$	9.3487	6.3456	8.79659	⋯	5.2476	0	8.8792
$f_{20}^{3}$	3.72586	7.0616	3.93986	⋯	5.0057	8.8792	0

## Appendix B. Ranked Matrices

**Table A2:** Ranked matrices for distance matrices disMATR 1 (top block, or rank 1), disMATR 2 (middle block, or rank 2), and disMATR 3 (bottom block, or rank 3).

	$f_{1}^{1}$	$f_{2}^{1}$	$f_{3}^{1}$	⋯	$f_{18}^{1}$	$f_{19}^{1}$	$f_{20}^{1}$
$f_{1}^{1}$	1	2	17	⋯	13	19	14
$f_{2}^{1}$	2	1	17	⋯	14	19	13
$f_{3}^{1}$	19	18	1	⋯	7	3	5
⋮	⋮	⋮	⋮	⋮	⋮	⋮	⋮
$f_{18}^{1}$	18	19	10	⋯	1	16	5
$f_{19}^{1}$	19	18	3	⋯	7	1	5
$f_{20}^{1}$	19	18	8	⋯	3	14	1
	$f_{1}^{2}$	$f_{2}^{2}$	$f_{3}^{2}$	⋯	$f_{18}^{2}$	$f_{19}^{2}$	$f_{20}^{2}$
$f_{1}^{2}$	1	16	9	⋯	6	3	20
$f_{2}^{2}$	16	1	5	⋯	12	10	9
$f_{3}^{2}$	10	4	1	⋯	9	5	20
⋮	⋮	⋮	⋮	⋮	⋮	⋮	⋮
$f_{18}^{2}$	6	16	13	⋯	1	11	20
$f_{19}^{2}$	4	16	7	⋯	6	1	20
$f_{20}^{2}$	20	6	8	⋯	14	17	1
	$f_{1}^{3}$	$f_{2}^{3}$	$f_{3}^{3}$	⋯	$f_{18}^{3}$	$f_{19}^{3}$	$f_{20}^{3}$
$f_{1}^{3}$	1	10	2	⋯	15	18	5
$f_{2}^{3}$	10	1	8	⋯	17	14	16
$f_{3}^{3}$	2	8	1	⋯	15	18	6
⋮	⋮	⋮	⋮	⋮	⋮	⋮	⋮
$f_{18}^{3}$	15	16	14	⋯	1	10	9
$f_{19}^{3}$	17	13	15	⋯	11	1	16
$f_{20}^{3}$	3	13	4	⋯	8	17	1
